# Future prospectives of sub-Saharan women living with HIV residing in France for more than seven years: prospective pilot study

**DOI:** 10.7448/IAS.17.4.19602

**Published:** 2014-11-02

**Authors:** Svetlane Dimi, Dominique Albucher, David Zucman

**Affiliations:** 1 Internal Medicine, Hopital Foch, Suresnes, France; 2 Service Social, Hopital Foch, Suresnes, France

## Abstract

**Introduction:**

In 2011, a French national survey of people living with HIV (PLHIV) has shown that 40% of persons diagnosed since 2003 are originated from sub-Saharan Africa, two thirds of them being women. For them, in the short term, access to social rights is a priority. Today, over 90% of PLHIV are treated effectively and with the aging of this population, questions about their future perspectives arise. Our service provides a multidisciplinary (medical, psychological, social) approach to PLHIV. The aim of our study is to describe the future perspectives of sub-Saharan women living with HIV residing in France for more than 7 years, because it is the time required for the implementation of fundamental rights and social insertion. Do they plan to return to their country of origin after their retirement? Does the HIV infection force them to stay in France?

**Materials and Methods:**

Prospective pilot mono-centric study. Between January and April 2014, every HIV-infected woman born in a sub-Saharan country, resident in France for at least 7 years, attending for their routine outpatient visit was consecutively included. Data were collected through a structured, semi-directed interview made by their usual hospital physician or social worker.

**Results:**

Consecutively, 76 women agreed to participate to the interview, none refused. Mean age: 42 years [26–70], time since HIV diagnosis: 12 years [1–25]. HIV diagnosis was made before arriving in France for 3% of them; in 33% diagnosis was made in the year of arrival; diagnosis made several years after arrival in 63%. Even if 69% of these women had been irregularly residing in France for a period, all of them had obtained a regular situation for residence and 50% acquired the French nationality. Mean duration of residence was 15 years [7–33]. Two thirds of them are employed. In the future, although 50% plan to have a shared residence between France and Africa, only 20% of them plan to settle back definitely in Africa and no woman declared that she would look for a medical follow up in Africa for their HIV infection.

**Conclusions:**

This study shows a good integration in France of HIV-infected sub-Saharan woman. Their links with Africa remain strong but very few plan to return in their country of origin due to lack of confidence in the African health infrastructures.

